# Contacting out-of-hours primary care or emergency medical services for time-critical conditions - impact on patient outcomes

**DOI:** 10.1186/s12913-019-4674-0

**Published:** 2019-11-07

**Authors:** Morten Breinholt Søvsø, Morten Bondo Christensen, Bodil Hammer Bech, Helle Collatz Christensen, Erika Frischknecht Christensen, Linda Huibers

**Affiliations:** 1 Centre for Prehospital and Emergency Research, Aalborg University, Søndre Skovvej 15, 9000 Aalborg, Denmark; 2 Research Unit for General Practice, Aarhus University, Aarhus, Denmark; 3 Department of Public Health, Research Unit of Epidemiology, Aarhus University, Aarhus, Denmark; 4 Emergency Medical Services, Copenhagen, University of Copenhagen, Copenhagen, Denmark; 5 Emergency Medical Services, North Denmark Region, Aalborg, Denmark

**Keywords:** Out-of-hours medical care, Delivery of healthcare, Primary care, Emergency medical services, Denmark, Myocardial infarction, Stroke, Sepsis, Telephone hotlines

## Abstract

**Background:**

Out-of-hours (OOH) healthcare services in Western countries are often differentiated into out-of-hours primary healthcare services (OOH-PC) and emergency medical services (EMS). Call waiting time, triage model and intended aims differ between these services. Consequently, the care pathway and outcome could vary based on the choice of entrance to the healthcare system.

We aimed to investigate patient pathways and 1- and 1–30-day mortality, intensive care unit (ICU) stay and length of hospital stay for patients with acute myocardial infarction (AMI), stroke and sepsis in relation to the OOH service that was contacted prior to the hospital contact.

**Methods:**

Population-based observational cohort study during 2016 including adult patients from two Danish regions with an OOH service contact on the date of hospital contact. Patients <18 years were excluded. Data was retrieved from OOH service databases and national registries, linked by a unique personal identification number. Crude and adjusted logistic regression analyses were performed to assess mortality in relation to contacted OOH service with OOH-PC as the reference and cox regression analysis to assess risk of ICU stay.

**Results:**

We included 6826 patients. AMI and stroke patients more often contacted EMS (52.1 and 54.1%), whereas sepsis patients predominately called OOH-PC (66.9%). Less than 10% (all diagnoses) of patients contacted both OOH-PC & EMS. Stroke patients with EMS or OOH-PC & EMS contacts had higher likelihood of 1- and 1–30-day mortality, in particular 1-day (EMS: OR = 5.33, 95% CI: 2.82–10.08; OOH-PC & EMS: OR = 3.09, 95% CI: 1.06–9.01). Sepsis patients with EMS or OOH-PC & EMS contacts also had higher likelihood of 1-day mortality (EMS: OR = 2.22, 95% CI: 1.40–3.51; OOH-PC & EMS: OR = 2.86, 95% CI: 1.56–5.23) and 1–30-day mortality. Risk of ICU stay was only significantly higher for stroke patients contacting EMS (EMS: HR = 2.38, 95% CI: 1.51–3.75). Stroke and sepsis patients with EMS contact had longer hospital stays.

**Conclusions:**

More patients contacted OOH-PC than EMS. Sepsis and stroke patients contacting EMS solely or OOH-PC & EMS had higher likelihood of 1- and 1–30-day mortality during the subsequent hospital contact. Our results suggest that patients contacting EMS are more severely ill, however OOH-PC is still often used for time-critical conditions.

## Background

In most Western countries, several healthcare services are available for out-of-hours healthcare (OOH), often differentiated into out-of-hours primary healthcare services (OOH-PC) and emergency medical services (EMS). For OOH-PC, various models exist, whereas EMS models are more similar across countries [1]. Different OOH-PC models include GP-cooperatives (GPCs), individual general practitioners (GPs), GP rotation groups and more. Telephone triage is widely used with the aim to ensure the right help to the right patients at the right time, but many services are also freely accessible [2, 3].

In Denmark, all out-of-hours services (i.e. EMS and OOH-PC) use telephone triage [4]. Patients are prompted to contact EMS in life- or limb-threatening situations and OOH-PC in less urgent situations that cannot wait until their own GP is available. Call waiting time and triage model differ between these services (i.e. type of call-handler and triage tools) as well as the intended aims of the services. Consequently, the care pathway and outcome could vary based on the choice of entrance to the healthcare system. If patients with time-critical conditions choose to contact OOH-PC, they may face a treatment delay with potential serious consequences [5, 6].

Time-critical conditions cover a diverse group of conditions, where fast medical intervention is crucial for the best outcome. Some time-critical conditions (e.g. acute myocardial infarction and stroke) often present with characteristic alarm symptoms [7, 8], whereas other conditions (e.g. sepsis) present with a variety of symptoms that may not lead to recognition of the severity or urgency of the situation [9].

Earlier studies have shown that contacting primary care services rather than EMS with symptoms of acute myocardial infarction or stroke increases risk of delayed treatment [5, 6], but only few smaller studies included patient-related clinical outcome measures such as differences in mortality or disease severity [10, 11]. Our objective was to investigate patient pathways and differences in patient-related clinical outcome measures (i.e. 1- and 1–30-day mortality, intensive care unit (ICU) stay and length of hospital stay) in patients with acute myocardial infarction, stroke and sepsis in relation to the OOH service that was contacted prior to hospital contact.

## Methods

### Study design and participants

We conducted a population-based observational cohort study from January 1st 2016 to December 31st 2016 including patients from two Danish regions with a contact to an OOH service on the date of hospital contact for acute myocardial infarction, stroke or sepsis. Diagnoses were identified according to the International Statistical Classification of Diseases and Health related Problems 10th Revision (ICD-10) [12]. See Additional file 1 for details and ICD-10 codes. Our sepsis definition was based on a previously published definition containing a number of selected ICD-10 codes [13] and our stroke definition included both hemorrhagic and ischemic stroke. Patients were only included with their first contact if they had more than one hospital contact during the study period. Other inclusion criteria were: minimum 18 years old, residing in one of the two regions, having a valid personal identification number (PIN), and having a contact outside office hours (as the OOH services had different opening hours). This study used the unique 10-digit PIN [14] for linkage to national registries (i.e. identifying hospital contacts with the diagnoses of interest [15]) and the OOH service databases (i.e. identifying whether the patient called OOH-PC and/or EMS [16]). Results are reported according to STROBE guidelines [17, 18].

### Setting

Two regions were selected to include patients from three types of OOH services. The North Denmark Region is a mixed rural and urban region with a population of 587,000 inhabitants [19] and the OOH services available are EMS and GPC. In the urban Capital Region of Copenhagen with 1.8 million inhabitants [19], the OOH services available are EMS and the Medical Helpline 1813. GPC and MH-1813 are both considered as OOH primary care.

Medical emergency calls to the national emergency number 1-1-2 are forwarded to the regional EMS, when health-related. Primarily nurses answer the calls, using a criteria-based dispatch protocol to assess the urgency and severity of the situation and the appropriate response (e.g. telephone advice, ambulance, paramedics, doctors) [20, 21]. EMS operate in a similar fashion in all five Danish regions. At the GPC, GPs answer all calls, performing triage and assessing the appropriate response (i.e. telephone advice, consultation, home visit or direct referral to hospital) [22]. Nurses (for the most part) and physicians answer the telephone at the Medical Helpline 1813 to decide whether the patient is in need of a telephone advice, consultation, a home visit, or a direct referral to the hospital [23]. The nurses use a decision support tool. Danish healthcare is tax-financed and free of charge, including the OOH services.

### Exposure, outcome measures and potential confounders

We defined the patients’ choice to contact a specific OOH service (i.e. OOH-PC, EMS or both EMS & OOH-PC) as the exposure in the present study. For each hospital contact considering the three time-critical conditions, we examined if an OOH service had been contacted on the same date and which service(s). This data was retrieved from the National Health Service Registry [16] and the OOH service databases.

Our primary outcome was defined as mortality 1 and 1–30 days after the hospital contact. Vital status was retrieved from the Civil Registration System [14].

Secondary outcomes were defined as probability of ICU stay during hospital stay and length of hospital stay associated with the contacted OOH service. This information was retrieved from the Danish National Patient Registry [15].

The association between exposure and outcome measures in this study could be confounded by patient characteristics (i.e. age, gender, ethnicity, employment status, income, education length and comorbidity). These factors have been found to relate to patient’s help-seeking behaviour and choice of entrance [24] as well as to mortality [25, 26]. Information on potential confounders was retrieved from Statistics Denmark [27] and the Danish National Patient Registry (i.e. diagnoses from past 5 years to determine comorbidity according to the Charlson Comorbidity Index [26, 28]).

### Statistical analysis

Data were anonymized for statistical analysis. Descriptive statistics were used for reporting population baseline characteristics, distribution of contacts to OOH services as well as length of stay.

Odds ratios (ORs) for 1- and 1–30-day mortality were calculated using logistic regression analyses. OOH service contact was the independent variable of primary interest. Income level was divided into quantiles based on the income level range in our population. Cox regression analysis was used to determine likelihood of ICU stay during hospital stay (hazard ratio (HR)) between OOH services for each of the time-critical conditions. Both crude and adjusted (for age, gender, ethnicity, income level, employment status, education length and comorbidity) analyses were performed for all analyses. The adjustment did not substantially change the results, therefore crude results are presented in the main text. However, results of the adjusted analyses can be seen in the appendix (Additional file 2).

We performed additional sensitivity analysis using the patient’s last contact (rather than the first contact) during the study period. This did not lead to any noteworthy changes as shown in Additional file 3. Kaplan-Meier survival curves were also computed to visualize differences in mortality in relation to OOH service. Results presented with 95% confidence intervals (CI), when relevant. Statistical analyses were performed with Stata V.15.0/MP (Stata Corporation, College Station, Texas, USA).

## Results

### Population

**Fig. 1:**
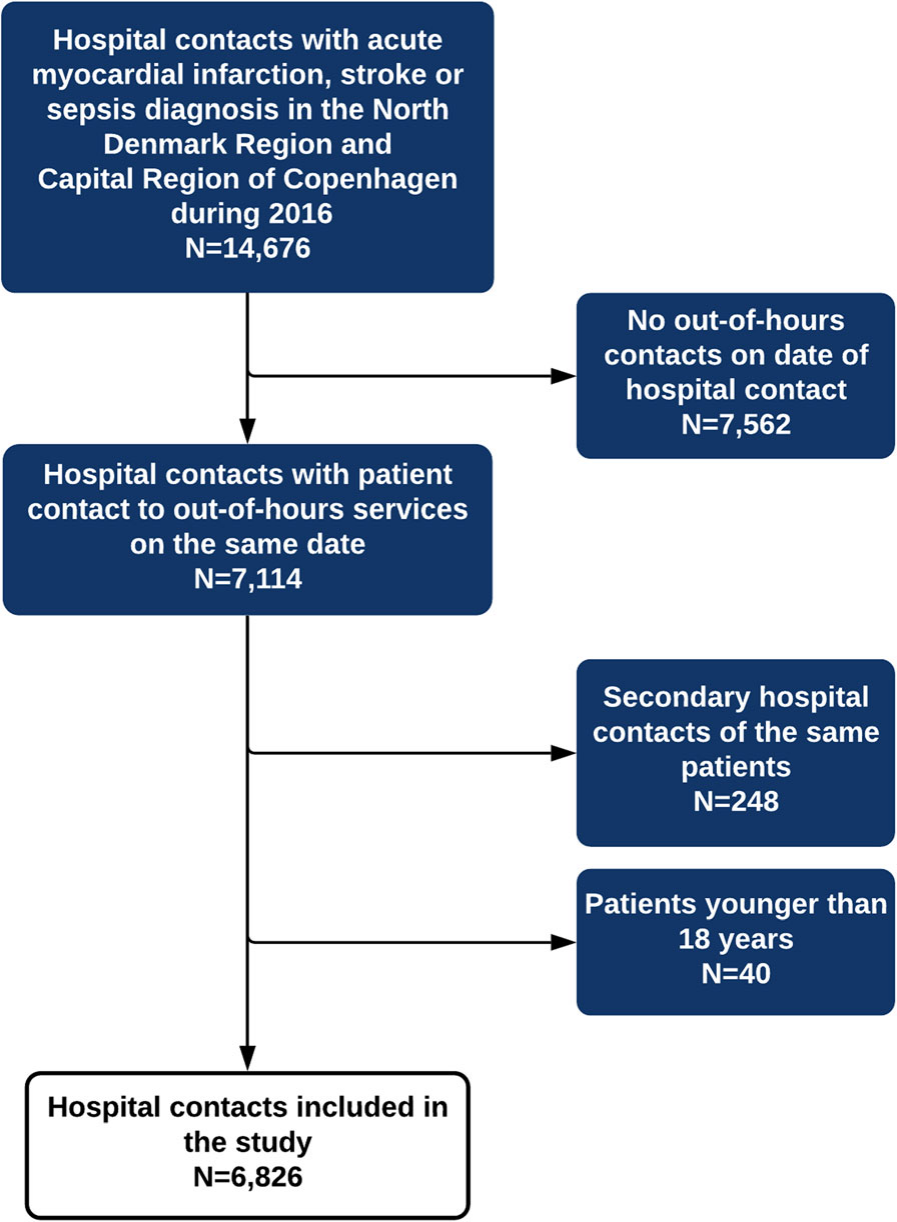
Flowchart showing the inclusion of the study population

In the North Denmark Region and the Capital Region of Copenhagen, 7114 admissions comprised the diagnoses of interest and had a registered contact to OOH services on the date of hospital contact during 2016. Only first hospital contacts were included in the study resulting in 6826 patients (Fig. 1).

Patients contacting OOH-PC or EMS had similar mean age (70.2 years (95% CI: 69.7–70.8) vs. 70.8 years (95% CI: 70.3–71.3)). OOH-PC contacts concerned women in 43.1% of the cases vs. 42.0% for EMS (Table 1). Additional population characteristics can be seen in Table 1 stratified by the OOH service contacted.

**Table 1:** Population baseline characteristics stratified by OOH service (N = 6826) (n, (%))

	OOH-PC	EMS	OOH-PC & EMS
Number	3401	2903	522
Age, mean, (95% CI)	70.2 (69.7–70.8)	70.8.1 (70.3–71.3)	71.6 (70.3–72.8)
Female gender	1464 (43.1)	1220 (42.0)	210 (40.2)
Employment status			
Employed	743 (21.9)	579 (19.9)	91 (17.4)
Unemployed (retired, on benefits, under education etc.)	2658 (78.2)	2324 (80.1)	431 (82.6)
Ethnicity			
Danish	3110 (91.4)	2642 (91.0)	488 (93.5)
Western countries	101 (3.0)	91 (3.1)	14 (2.7)
Non-western countries	190 (5.6)	170 (5.9)	20 (3.8)
Education length^a^			
<=10 years	1404 (41.3)	1180 (40.6)	237 (45.4)
>10- ≤ 15 years	1380 (40.6)	1250 (43.1)	214 (41.0)
>15 years	617 (18.1)	473 (16.3)	71 (13.6)
Income level (quantiles)			
1 (low)	727 (21.4)	739 (25.5)	127 (24.3)
2	922 (27.1)	815 (28.1)	156 (29.9)
3	849 (25.0)	693 (23.9)	148 (28.4)
4 (high)	903 (26.6)	656 (22.6)	91 (17.4)
Charlson Comorbidity Index (CCI)			
CCI 0	1824 (53.6)	1624 (55.9)	282 (54.0)
CCI 1–2	1121 (33.0)	916 (31.6)	178 (34.1)
CCI > =3	456 (13.4)	363 (12.5)	62 (11.9)

^a^ < =10 years (primary school), > 10–15 years (vocational educations, gymnasium, short-cycle higher education), > 15 years (medium-cycle higher education, long-cycle higher education, university)

**Fig. 2:**
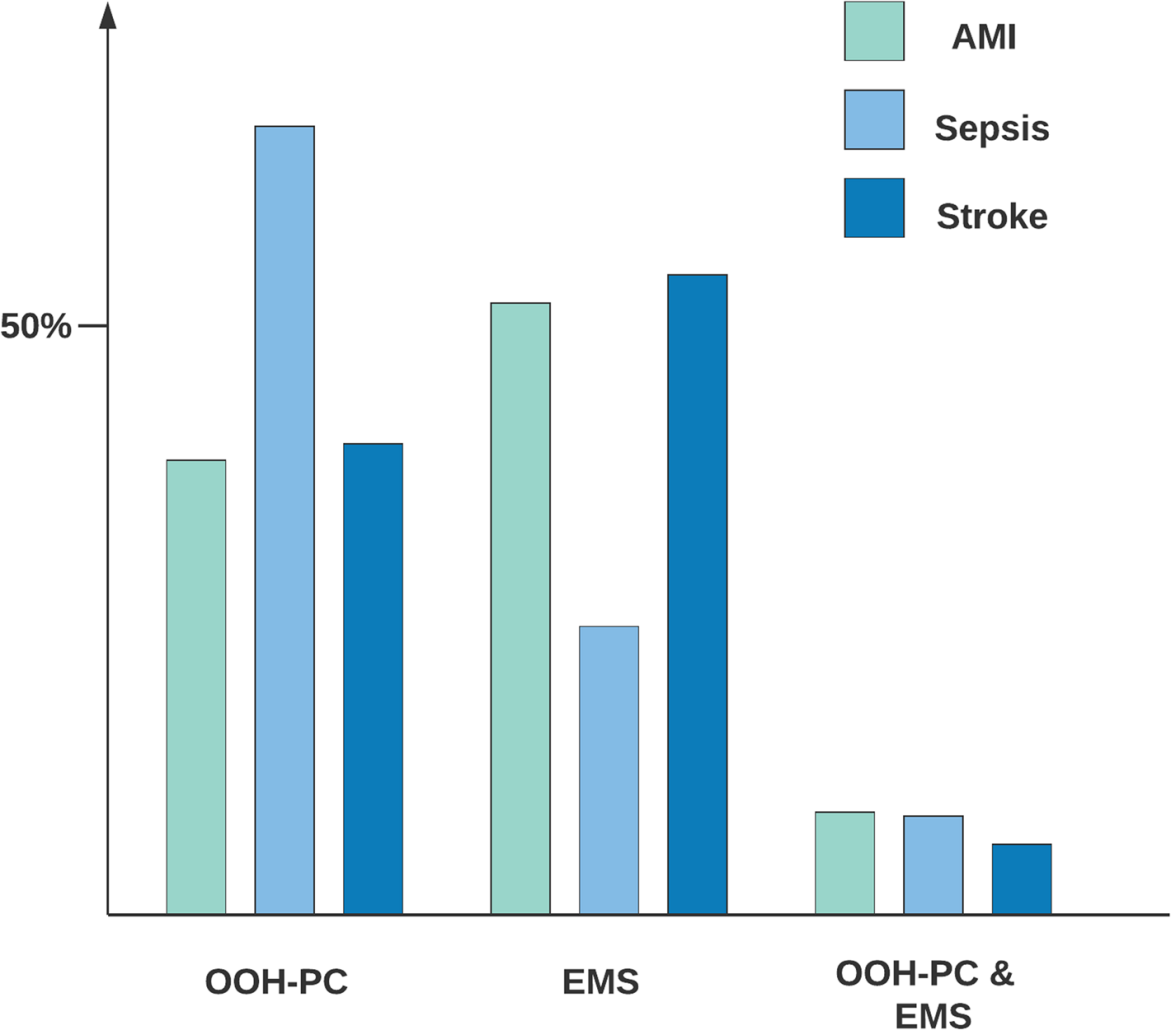
Distribution of OOH services contacted prior to hospital contact for the included conditions in percent (*N* = 6826)

OOH-PC handled 49.8% of all included patients and EMS 42.5%, whereas 7.6% had contacts to both OOH-PC & EMS. EMS handled the majority of AMI patients (52.1%) (Fig. 2), while 39.2% had a contact with OOH-PC. Two-thirds of all sepsis patients (66.9%) solely had contact with OOH-PC on the date of hospital contact. Stroke patients were predominately handled by EMS (54.1%) followed by OOH-PC (39.9%). Patients with stroke included both hemorrhagic (21.3%) and ischemic stroke (78.7%). Their pathway differed as 65.3% of patients with hemorrhagic stroke contacted EMS compared to 51.1% of patients with ischemic stroke (Additional file 4).

### Primary outcome - mortality

**Fig. 3:**
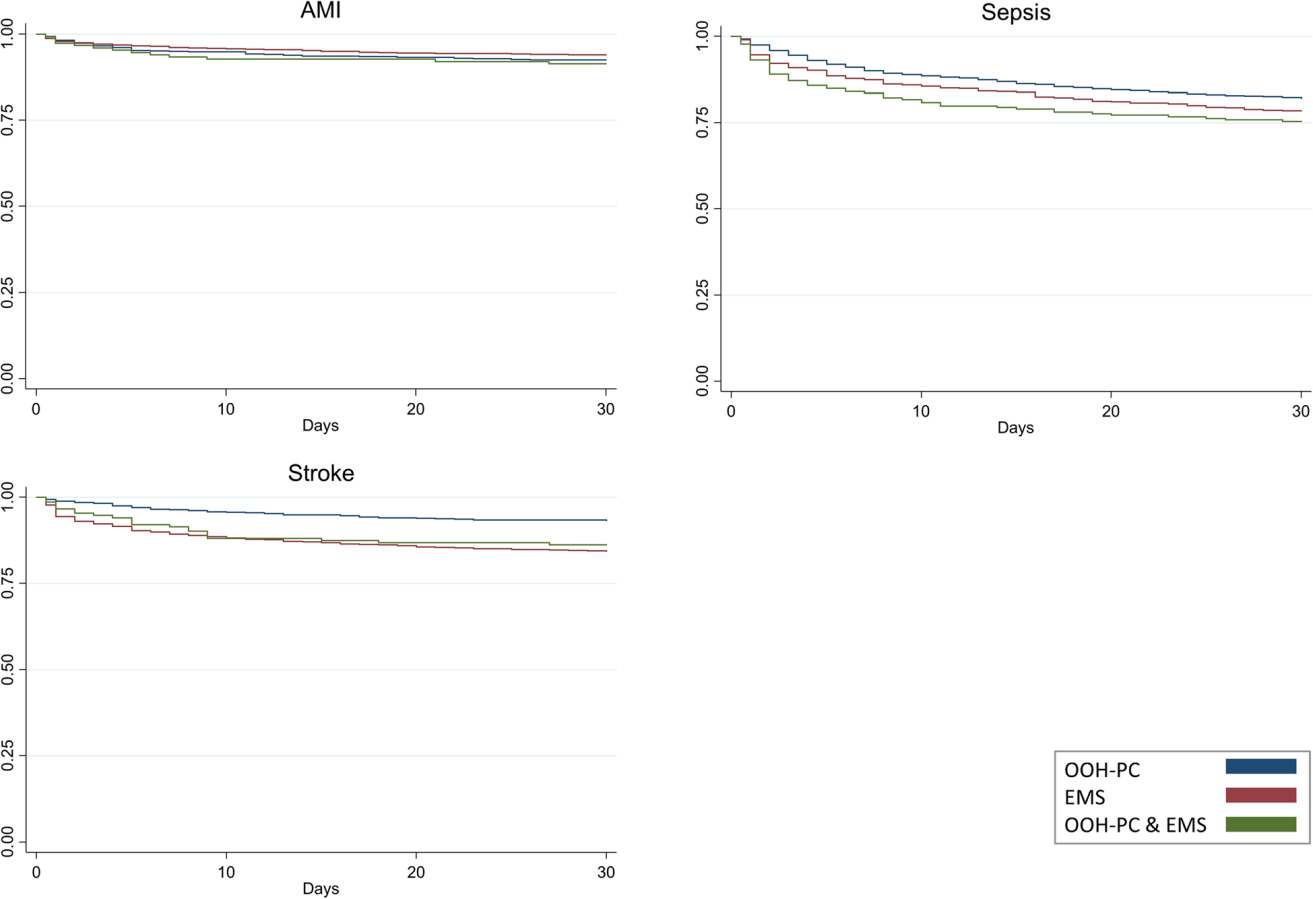
Kaplan-Meier survival curves for AMI, sepsis and stroke stratified by OOH service (N = 6826)

As illustrated by the Kaplan-Meier survival curves (Fig. 3), mortality was high in the first 24 h after hospital contact for patients with AMI, stroke and sepsis. Patients with AMI displayed no evident differences in mortality on the basis of the OOH service, whereas both stroke and sepsis patients displayed higher mortality after EMS contact or OOH-PC & EMS contact throughout the 30 days studied compared to OOH-PC contact alone. Mortality in percent for the included conditions can be seen in Table 2.

**Table 2:** Crude analysis of the association between OOH service prior to contact, 1- and 1–30-day mortality and ICU stay (*N* = 6826)

Diagnosis	Service	1-day mortality		1–30-day mortality		Intensive care unit stay	
	N	N (%)	OR (95% CI)	N (%)	OR (95% CI)	N (%)	HR (95% CI)
AMI (*N* = 1734)	OOH-PC (679)	12 (1.77)	ref	51 (7.51)	ref	12 (1.77)	ref
	EMS (904)	19 (2.10)	1.29 (0.58–2.48)	54 (5.97)	0.78 (0.53–1.16)	29 (3.21)	1.66 (0.85–3.27)
	OOH-PC & EMS (151)	<5 (NR^a^)	1.51 (0.48–4.76)	13 (8.61)	1.16 (0.61–2.19)	<5 (NR^a^)	1.44 (0.46–4.49)
Sepsis (*N* = 2561)	OOH-PC (1713)	43 (2.51)	ref	308 (17.98)	ref	42 (2.45)	ref
	EMS (629)	34 (5.41)	2.22 (1.40–3.51)	136 (21.62)	1.26 (1.00–1.58)	39 (6.20)	1.56 (0.99–2.46)
	OOH-PC & EMS (219)	15 (6.85)	2.86 (1.56–5.23)	54 (24.66)	1.49 (1.07–2.08)	8 (3.65)	1.14 (0.53–2.43)
Stroke (*N* = 2531)	OOH-PC (1009)	11 (1.09)	ref	68 (6.74)	ref	23 (2.28)	ref
	EMS (1370)	76 (5.55)	5.33 (2.82–10.08)	214 (15.62)	2.56 (1.92–3.41)	110 (8.03)	2.38 (1.51–3.75)
	OOH-PC & EMS (152)	5 (3.29)	3.09 (1.06–9.01)	21 (13.82)	2.22 (1.32–3.74)	7 (4.61)	1.94 (0.83–4.53)

^a^*NR* not reported due to too few observations

No significant differences in odds for 1- nor 1–30-day mortality for AMI patients in relation to OOH service were found (Table 2). On the contrary, stroke patients had a higher likelihood of 1- and 1–30-day mortality, when contacting EMS alone or OOH-PC & EMS compared to OOH-PC, in particular 1-day mortality (EMS: OR = 5.33, 95%CI: 2.82–10.08; OOH-PC & EMS: OR = 3.09, 95%CI: 1.06–9.01). Within the stroke group, patients with hemorrhagic stroke had substantially higher mortality than patients with ischemic stroke, especially around day 1 (Additional file 5). Patients who contacted EMS alone or OOH-PC & EMS prior to a hospital contact for sepsis also had a higher likelihood of 1-day mortality (EMS: OR = 2.22. 95%CI: 1.40–3.51; OOH-PC & EMS: OR = 2.86. 95%CI: 1.56–5.23) and 1–30-day mortality as well (EMS: OR = 1.26 95%CI: 1.00–1.58; OOH-PC & EMS: OR = 1.49 95%CI: 1.07–2.08).

### Secondary outcomes – ICU stay and length of stay

**Fig. 4:**
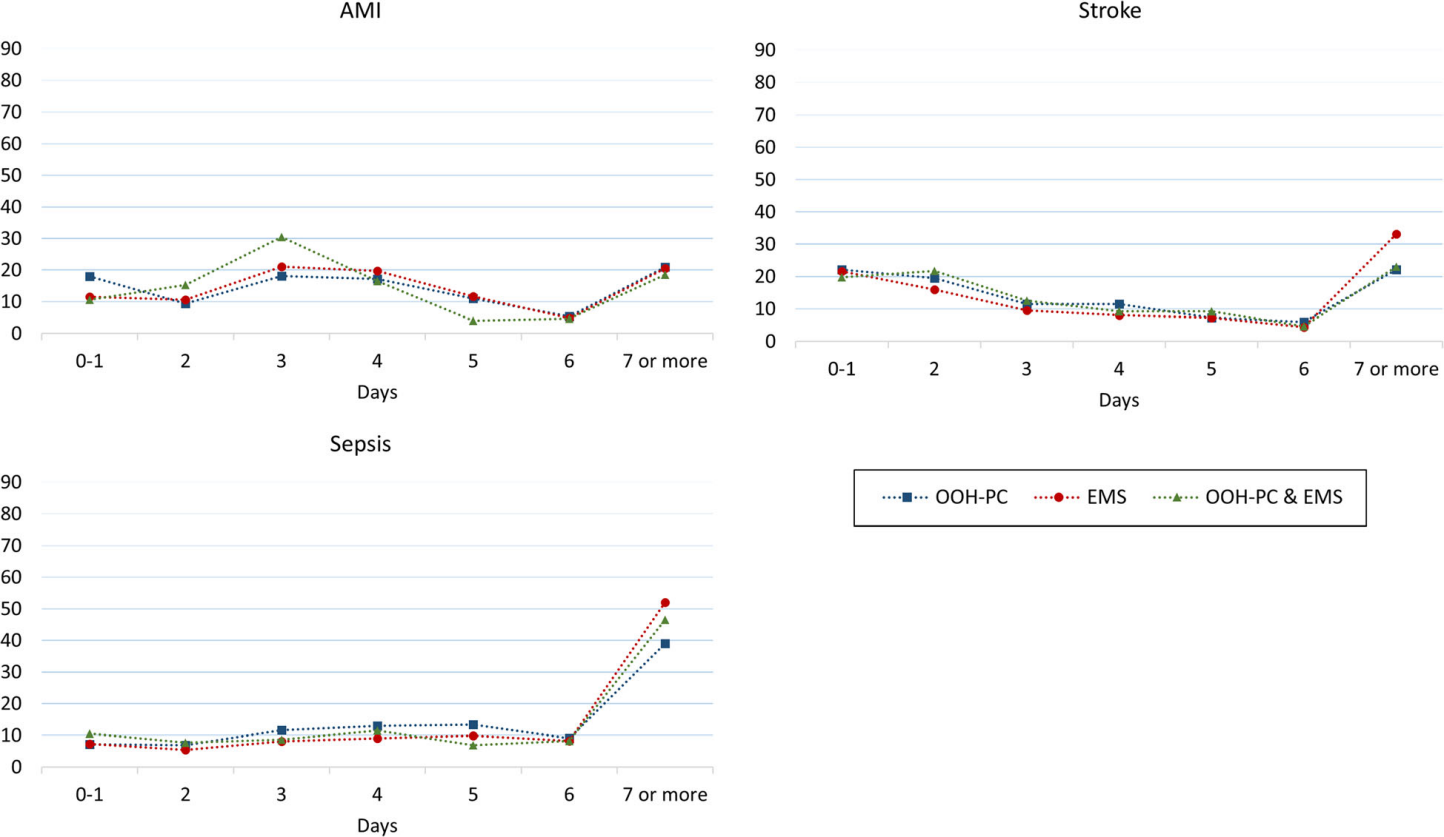
Length of hospital stay for all conditions shown as percentage of all OOH contacts within each service (*N* = 6826)

Regardless of the diagnosis, patients contacting EMS showed a tendency towards increased risk of ICU stay compared to patients contacting OOH-PC (Table 2). However, this association was only statistically significant for stroke patients. Patients with AMI and OOH-PC contacts had more one-day hospital stays, whereas more stroke and sepsis patients with EMS contacts had longer hospital stays (Fig. 4).

## Discussion

### Key results

In almost half of OOH hospital contacts with the three included time-critical conditions, patients contacted OOH-PC. In addition, more than two-thirds of patients with sepsis solely contacted OOH-PC prior to hospital contact.

Contacting the EMS or OOH-PC & EMS prior to admission for stroke or sepsis showed higher likelihood of 1- and 1–30-day mortality compared to contacting OOH-PC – in particular 1-day mortality. As expected, EMS contacts prior to hospital contact displayed a tendency towards higher risk of ICU stays. Hospital contacts with stroke or sepsis following EMS contacts more often resulted in longer hospital stays compared to OOH-PC contacts.

### Strengths and weaknesses of the study

The present study investigated the impact of the choice of contacting an OOH service in case of a time-critical condition on patient outcomes, including all available OOH services and a large cohort. Furthermore, the study has a population-based design, which minimized selection bias, as every hospital contact of patients within two regions with the diagnoses of interest were included. This was made possible through the unique PIN, which also allowed for extensive registry linkage (including sociodemographic data), linkage to OOH services and complete follow-up.

The disease groups in the present study were compiled by relevant ICD-10 diagnoses, which entailed two limitations: diagnoses included may vary when comparing to other studies and no other clinical data was obtained to verify the diagnoses. However, the validity of the Danish National Patient Registry is relatively high (positive predictive values range for AMI: 81.9–100 (I24 not included), stroke: 71.8–97.0 (similar definition), sepsis: 21.7–85.7 (definition varies)) [29]. We based the inclusion of relevant ICD-10 codes for sepsis on an earlier study to include as many relevant diagnoses as possible, but this study also found that sepsis is underreported in Danish registries [13]. Consequently, we have most likely missed some patients with sepsis in hospital. In addition, an earlier study found increasing completeness of sepsis registration with increasing severity of the patient’s condition [30]. If this is the case in our cohort, we will have missed patients with less severe conditions. The majority of sepsis patients contacted OOH-PC in our study and we would also expect patients with less severe conditions to do so. If these missing patients were included, this would not change our message of patients with contacts to EMS being more severely ill compared to OOH-PC contacts prior to hospital contact. Our stroke group included both hemorrhagic and ischemic stroke. Although other studies have done the same [11, 31], combining the two may level out associations between stroke subtype and outcome measures. Furthermore, a number of contacts to the OOH services did not have a registered PIN, primarily at the EMS. Consequently, we may have missed some patient contacts with the EMS prior to hospital contact, which implies a risk of selection bias. Missing PIN have been shown to be an issue in the least urgent EMS contacts [32] and is known to occur in contacts with very high urgency. This may have affected our cohort size, but not likely our results as the high and low urgency would level out each other in the association with outcome. However, our study might have been limited by possible data loss regarding hospital contacts due to implementation of new electronic medical records in the hospitals in the Capital Region of Copenhagen. Thus, the number of patients with the conditions of interests might be underestimated. However, the data loss was a general problem not related to which OOH service was used, thus we have no reason to believe it influences our outcome measures. We may have underestimated the group of patients that have contacts to OOH-PC & EMS as well as patients calling just before midnight with a subsequent hospital contact the following date, since we based our method on dates and not on time-intervals measured as hours. Most likely this would only affect cohort size and not the results. Lastly, the association between exposure and outcome measures in this study could be confounded by other key variables than patient characteristics (e.g. emergency department crowding, hospital characteristics [33–35]), which we did not have access to. Lack of this information may have led to an overestimation of the association between choice of OOH service and our outcome measures.

### Comparison with literature

Studies from Western countries on time-critical conditions in OOH services are dominated by time-to-treatment and components-of-delay studies – especially regarding AMI, closely followed by sepsis and stroke [5, 6, 11, 31, 36, 37]. However, some of these earlier studies have also investigated the patient pathway for certain time-critical conditions. Studies investigating acute coronary syndrome/STEMI found that the proportion of contacts to primary care (not specifically OOH-PC) as the first medical contact ranged from 14 to 47.5% of included cases. In similar studies investigating stroke patients, the number ranged from 36.1 to 49.4%. The majority of these studies found that contacting primary care increased prehospital delay, which was most often defined as the time from symptom onset to arrival at hospital. Nevertheless, patient delay (from symptom onset to healthcare contact) was often quite substantial for patients who chose to contact primary care when compared to EMS. Among the studies of stroke patients, only one reported patient outcome. This study by Faiz et al. found milder neurologic deficits in patients calling primary care compared to patients calling EMS [11], still mortality was not reported.

Loots et al. [38] investigated 263 sepsis patients admitted to the ICU with (48.3%) and without GP contact, whereas Latten et al. [10] investigated 440 adult emergency department patients with infections or suspected infections comparing GP referred patients (83%) with EMS patients. No significant differences in mortality was found among patients with or without a GP contact, not unlikely due to study sizes. Nevertheless, Latten et al. did find that GP-referred patients were less often triaged with high urgency and admitted to the ICU.

Our results indicate that patients with more severe disease contacted EMS to a greater extent, possibly due to self-triage, suggesting that patients may be able to choose the best fit OOH service. On the other hand a large proportion of patients with AMI and stroke – conditions that often present with alarming symptoms – contacted OOH-PC. Two studies of patients with suspected AMI not calling an ambulance reported that non-callers were less likely to have an AMI and fewer had a history of ischemic heart disease [39, 40]. Not feeling critically ill was the main reason reported for not calling an ambulance, nevertheless 46 and 10% of non-callers had a confirmed AMI in the two studies, perhaps due to poor understanding of symptoms and/or severity of the condition. Patients’ evaluation of their own health is only one part of help-seeking behavior - a complex concept comprised of cultural, social, economic, geographical and organizational determinants [41, 42]. Some of these determinants have been investigated in relation to seeking OOH healthcare. Age, ethnicity, low education, unemployment and history of frequent healthcare contacts were associated with higher likelihood of contacting OOH service, whereas no or little social support and/or a high health literacy level was associated with less likelihood of using OOH [24].

### Implications for practice and future research

Although the conditions AMI and stroke often present with alarming symptoms, 40% of these patients contacted OOH-PC and not EMS. Furthermore, patients contacting OOH-PC & EMS were at risk of poor outcome, thus additional public information on when a situation is urgent and how to utilize the OOH system is necessary. In addition, organization of the OOH services could be adjusted to match patient behavior and need, when calling either the acute or non-acute number. Improving the collaboration of the OOH services or creating a more seamless transition between OOH-PC and EMS may aid the patient when contacting healthcare, as the possibility of redirecting the patient to the best fit OOH service would be improved for the healthcare personnel. This could be through compatible telephone systems and medical record systems accessible to both OOH-PC and EMS and perhaps co-location of call centers. Furthermore, hospital healthcare personnel should be aware that patients referred directly from OOH-PC may still be severely ill and that double contact patients seem to be a risk group in need of special attention. Future research should focus on patients with double contacts, to get more insight in their care pathway and symptom presentation. Also, the possibility of establishing more collaboration between OOH services should be studied.

## Conclusion

With this study, we aimed to investigate whether patients choose the OOH service best fit to handle their condition. We expected EMS patients to be more severely ill than OOH-PC patients, since the aim of EMS is to provide care to patients with life-threatening conditions. Compared to patients contacting OOH-PC prior to hospital contacts, stroke and sepsis patients contacting EMS only or OOH-PC & EMS had higher likelihood of 1- and 1–30-day mortality, a tendency towards higher likelihood of ICU stay and more often longer hospital stays. Nevertheless, we found that the nearly half of patients with the included time-critical conditions contacted OOH-PC.

## Supplementary information

**Additional file 1.** ICD-10 codes included in study population. List of all included ICD-10 codes in the study.

**Additional file 2. **Adjusted analysis of the association between OOH service, ICU stay and mortality (*N* = 6826). Analysis of the association between OOH service, 1- and 1–30-day mortality and ICU stay. Adjusted for age, gender, ethnicity, employment status, education level, income level & comorbidity. * NR = not reported due to too few observations.

**Additional file 3. **Sensitivity analysis for the association between OOH service, ICU stay and mortality (*N* = 6826). Crude analysis of the association between OOH service, 1- and 1–30 day mortality and ICU stay using the patients’ last hospital contact during the study period.

**Additional file 4. **OOH services contacted prior to hospital contact within the stroke subgroups. Stroke subtypes (brain hemorrhage (*N* = 539) and stroke (*N* = 1996) and choice of OOH service prior to hospital contact.

**Additional file 5. **Differences in mortality for brain hemorrhage & ischemic stroke. Kaplan-Meier survival curve showing differences in mortality for stroke subtypes (brain hemorrhage (*N* = 539) and stroke (*N* = 1996).

## Data Availability

The data that support the findings of this study are available from the North Denmark Region and the Capital Region of Copenhagen, but restrictions apply to the availability of these data, which were used under license from the Danish Patient Safety Authority for the current study, and so are not publicly available.

## Abbreviations

AMI: acute myocardial infarction
CI: confidence intervals
EMS: Emergency Medical Services
GP: general practitioner
GPC: general practitioner cooperative
HR: hazard ratio
ICD-10: International Statistical Classification of Diseases, 10th Edition
ICU: intensive care unit
OOH: out-of-hours
OOH-PC: out-of-hours primary care
OR: odds ratio
PIN: personal identification number
STEMI: ST-elevation myocardial infarction
STROBE: STrengthening the Reporting of Observational Studies in Epidemiology
