# Supplementary material for: Contacting out-of-hours primary care or emergency medical services for time-critical conditions - impact on patient outcomes
Source: BMC Health Serv Res. 2019 Nov 7;19:813. doi: 10.1186/s12913-019-4674-0 (PMC6839230; doi:10.1186/s12913-019-4674-0)
Supplement: Supplementary file 1 — Additional file 1. ICD-10 codes included in study population. List of all included ICD-10 codes in the study. [file 12913_2019_4674_MOESM1_ESM.docx]

| **Diagnosis** | **ICD-10 codes included in study population** |
| --- | --- |
| **Acute myocardial infarction** | Acute myocardial infarction **I210** (I210A, I210B, I211, I211A, I211B, I213, I214, I219)  Other acute ischaemic heart diseases **I240** (I240A, I241, I248, I248A, I249) |
| **Stroke (incl. hemorrhagic stroke)** | Subarachnoid haemorrhage **I600** (I601, I602, I603, I604, I605, I606, I606A, I606B, I606C, I607, I607A, I608, I609, I609A)  Intracerebral haemorrhage **I610** (I611, I611A, I611B, I6612, I613, I614, I615, I616, I618, I619)  Other non-traumatic intracranial haemorrhage **I620** (I621, I629)  Cerebral infarction **I630** (I631, I632, I633, I634. I635, I636, I638, I639)  Stroke, not specified as haemorrhage or infarction **I640** (I649) |
| **Sepsis** | Salmonella sepsis **A021**  Septicaemic plague **A207**  Anthrax sepsis **A227**  Erysipelothrix sepsis **A267**  Extraintestinal yersiniosis **A282B**  Listerial sepsis **A327**  Acute meningococcaemia **A392** (A392A)  Meningococcaemia, unspecified **A394**  Streptococcal sepsis **A400** (A401, A402, A403, A408, A409)  Other sepsis **A410** (A411, A411A, A412, A413, A414, A415, A415A, A418, A419, A19B, A419C, A4127)  Bacteraemia, not otherwise specified **A499A**  Gonoccocal sepsis **A548G**  Candidal sepsis **B377**  Fungaemia, not otherwise specified **B499A** |
