# Supplementary material for: Contacting out-of-hours primary care or emergency medical services for time-critical conditions - impact on patient outcomes
Source: BMC Health Serv Res. 2019 Nov 7;19:813. doi: 10.1186/s12913-019-4674-0 (PMC6839230; doi:10.1186/s12913-019-4674-0)
Supplement: Supplementary file 2 — Additional file 2. Adjusted analysis of the association between OOH service, ICU stay and mortality (N = 6826). Analysis of the association between OOH service, 1- and 1–30-day mortality and ICU stay. Adjusted for age, gender, ethnicity, employment status, education level, income level & comorbidity. * NR = not reported due to too few observations. [file 12913_2019_4674_MOESM2_ESM.docx]

| Diagnosis | Service | 1-day mortality | | 30-day mortality | | Intensive care unit stay | |
| --- | --- | --- | --- | --- | --- | --- | --- |
|  |  | N (%) | OR* (95%CI) | N (%) | OR*(95%CI) | N (%) | HR* (95%CI) |
| AMI  (N=1,734) | OOH-PC (N=679) | 12 (1.77) | ref | 51 (7.51) | ref | 12 (1.77) | ref |
|  | EMS (N=904) | 19 (2.10) | 1.01 (0.48-2.14) | 54 (5.97) | 0.68 (0.45-1.03) | 29 (3.21) | 1.83 (0.92-3.68) |
|  | OOH-PC & EMS (N=151) | <5 (NR) | 1.34 (0.41-4.37) | 13 (8.61) | 1.05 (0.53-2.08) | <5 (NR) | 2.04 (0.64-6.51) |

| Sepsis  (N=2,561) | OOH-PC (N=1,713) | 43 (2.51) | ref | 308 (17.98) | ref | 42 (2.45) | ref |
| --- | --- | --- | --- | --- | --- | --- | --- |
|  | EMS (N=629) | 34 (5.41) | 2.09 (1.31-3.33) | 136 (21.62) | 1.17 (0.92-1.48) | 39 (6.20) | 1.52 (0.96-2.41) |
|  | OOH-PC & EMS (N=219) | 15 (6.85) | 2.53 (1.37-4.68) | 54 (24.66) | 1.31 (0.93-1.85) | 8 (3.65) | 1.19 (0.55-2.56) |

| Stroke  (N=2,531) | OOH-PC (N=1,009) | 11 (1.09) | ref | 68 (6.74) | ref | 23 (2.28) | ref |
| --- | --- | --- | --- | --- | --- | --- | --- |
|  | EMS (N=1,370) | 76 (5.55) | 5.29 (2.79-10.03) | 214 (15.62) | 2.63 (1.96-3.53) | 110 (8.03) | 2.37 (1.50-3.74) |
|  | OOH-PC & EMS (N=152) | 5 (3.29) | 3.26 (1.11 -9.60) | 21 (13.82) | 2.51 (1.45-4.33) | 7 (4.61) | 1.65 (0.69-3.94) |
