# Supplementary material for: Contacting out-of-hours primary care or emergency medical services for time-critical conditions - impact on patient outcomes
Source: BMC Health Serv Res. 2019 Nov 7;19:813. doi: 10.1186/s12913-019-4674-0 (PMC6839230; doi:10.1186/s12913-019-4674-0)
Supplement: Supplementary file 3 — Additional file 3. Sensitivity analysis for the association between OOH service, ICU stay and mortality (N = 6826). Crude analysis of the association between OOH service, 1- and 1–30 day mortality and ICU stay using the patients’ last hospital contact during the study period. [file 12913_2019_4674_MOESM3_ESM.docx]

| Diagnosis | Service | 1-day mortality | 30-day mortality | Intensive care unit stay |
| --- | --- | --- | --- | --- |
|  | N | OR (95%CI) | OR (95%CI) | HR (95%CI) |
| AMI  (N=1,727) | OOH-PC (684) | ref | ref | ref |
|  | EMS (894) | 1.28 (0.62-2.64) | 0.80 (0.54-1.18) | 1.71 (0.87-3.36) |
|  | OOH-PC & EMS (149) | 1.55 (0.49-4.87) | 1.24 (0.67-2.29) | 1.50 (0.48-4.66) |

| Sepsis  (N=2,587) | OOH-PC (1,741) | ref | ref | ref |
| --- | --- | --- | --- | --- |
|  | EMS (633) | 2.15 (1.37-3.77) | 1.28 (1.03-1.60) | 1.60 (1.02-2.50) |
|  | OOH-PC & EMS (213) | 2.99 (1.66-5.39) | 1.71 (1.24-2.36) | 1.19 (0.56-2.55) |

| Stroke  (N=2,512) | OOH-PC (1,003) | ref | ref | ref |
| --- | --- | --- | --- | --- |
|  | EMS (1,356) | 5.58 (2.95-10.54) | 2.65 (1.99-3.52) | 2.28 (1.47-3.53) |
|  | OOH-PC & EMS (153) | 3.05 (1.04-8.90) | 2.19 (1.30-3.69) | 1.76 (0.76-4.08) |
