# Supplementary figures and images for: Contacting out-of-hours primary care or emergency medical services for time-critical conditions - impact on patient outcomes
Source: BMC Health Serv Res. 2019 Nov 7;19:813. doi: 10.1186/s12913-019-4674-0 (PMC6839230; doi:10.1186/s12913-019-4674-0)

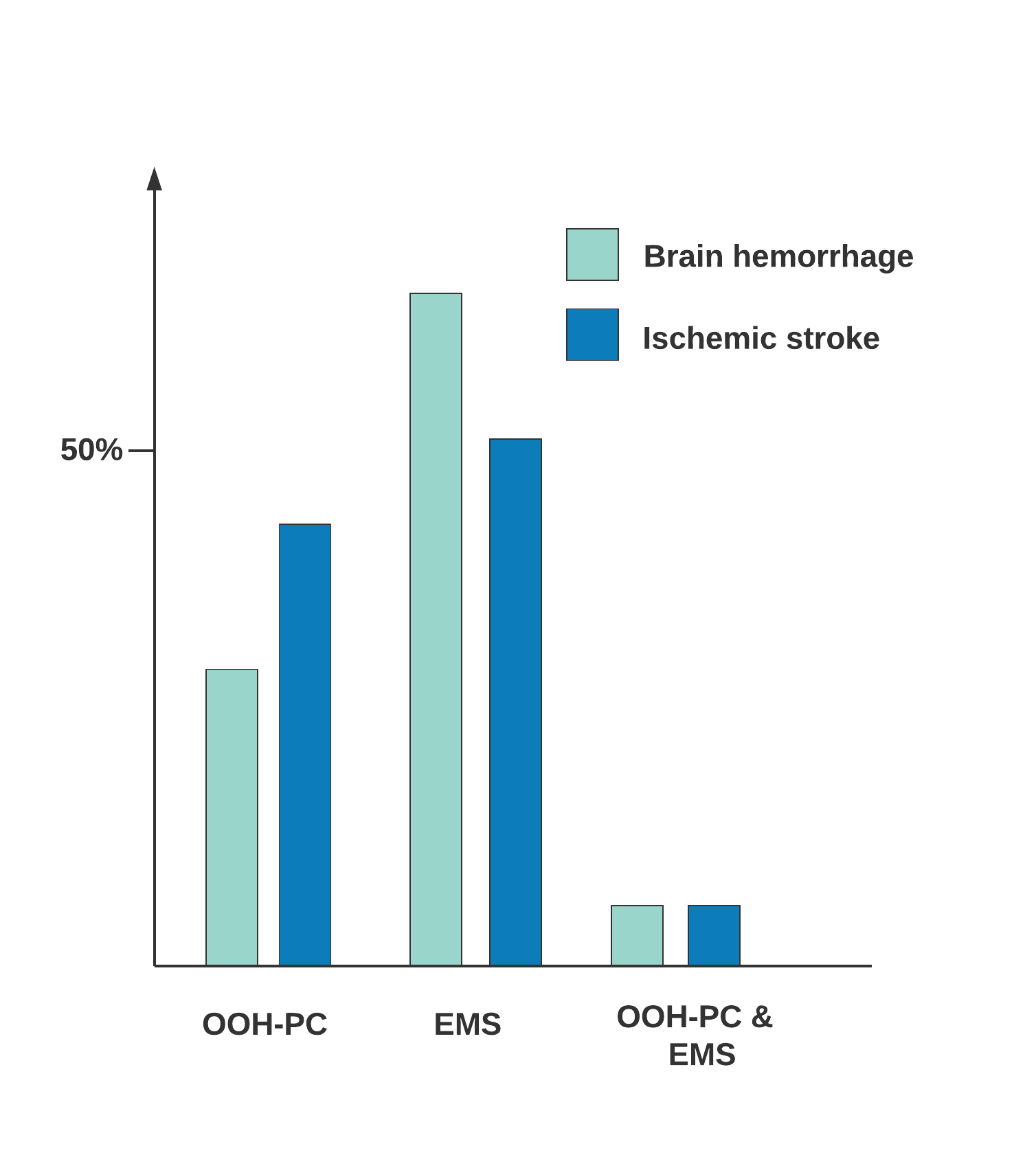

Supplement: Supplementary file 4 — Additional file 4. OOH services contacted prior to hospital contact within the stroke subgroups. Stroke subtypes (brain hemorrhage (N = 539) and stroke (N = 1996) and choice of OOH service prior to hospital contact. [file 12913_2019_4674_MOESM4_ESM.png]

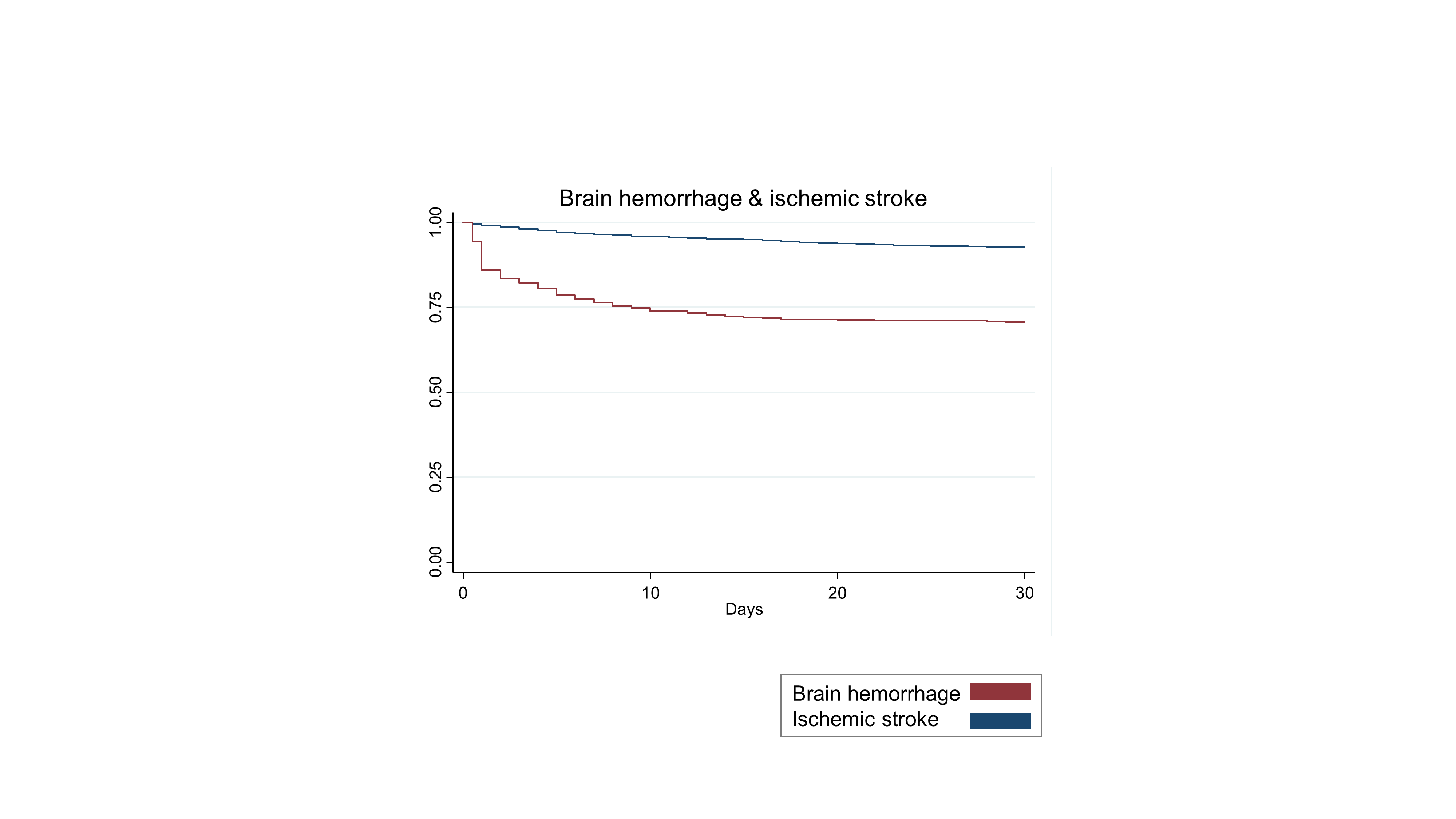

Supplement: Supplementary file 5 — Additional file 5. Differences in mortality for brain hemorrhage & ischemic stroke. Kaplan-Meier survival curve showing differences in mortality for stroke subtypes (brain hemorrhage (N = 539) and stroke (N = 1996). [file 12913_2019_4674_MOESM5_ESM.tif]
